# Regional Disparities and Determinants of Visual Impairment Among Diabetic Patients in Ethiopia

**DOI:** 10.1002/hsr2.71685

**Published:** 2025-12-21

**Authors:** Chalachew Yenew, Yehzibalem Azmeraw, Almaw Genet Yeshiwas

**Affiliations:** ^1^ Department of Public Health Debre Tabor University Debra Tabor Ethiopia; ^2^ Jockey Club College of Veterinary Medicine and Life Sciences City University of Hong Kong Hong Kong SAR China; ^3^ Department of Plant Science, College of Agriculture, Food and Climate Sciences Injibara University Injibara Ethiopia; ^4^ Department of Environmental Health, College of Medicine and Health Science Injibara University Injibara Ethiopia

**Keywords:** Ethiopia, geographic disparities, glycemic control, prevalence, risk factors, visual impairment (VI)

## Abstract

**Background:**

The increasing prevalence of diabetes in Ethiopia has led to a rising burden of its complications, particularly Visual Impairment (VI). This systematic review and meta‐analysis aimed to determine the pooled prevalence, identify associated risk factors, and evaluate geographic disparities of VI among diabetic patients in Ethiopia.

**Method:**

A comprehensive literature search was conducted across multiple electronic databases up to January 30, 2024. Data was extracted with an Excel Sheet and analyzed using RevMan 5.4 and **R statistical software (version 4.5.1)**. A random‐effects model was used to estimate the pooled prevalence and to perform subgroup analyses by geographic region and publication period. Risk factors were pooled to generate adjusted odds ratios (AORs).

**Results:**

The analysis included 11 studies with 4,204 diabetic patients. The overall pooled prevalence of VI was 29.32% (95% CI: 18.92%, 39.71%). Subgroup analysis revealed profound and statistically significant geographic disparities (Test for subgroup differences: χ² = 274.10, df = 4, *p* < 0.0001), with the prevalence ranging from 7% (95% CI: 0.05, 0.11) in Oromia to 70% (95% CI: 0.65, 0.75) in Tigray. The pooled estimates for Amhara and SNNP regions were 27% (95% CI: 0.18, 0.39) and 21% (95% CI: 0.11, 0.37), respectively. Key factors significantly associated with increased odds of VI were older age (60‐80 years; AOR = 3.55, 95% CI: 1.23, 10.28), diabetes duration > 3 years (AOR = 3.47, 95% CI: 2.43, 4.95), poor glycemic control (AOR = 3.18, 95% CI: 2.20, 4.59), and inadequate physical activity (AOR = 2.74, 95% CI: 1.89, 3.97).

**Conclusion and Recommendation:**

There is a high and geographically heterogeneous burden of visual impairment among diabetic patients in Ethiopia. The identified risk factors are largely modifiable. We recommend implementing region‐specific public health interventions that prioritize promoting physical activity, optimizing glycemic control, and managing diabetes duration effectively to mitigate the risk and disparity of visual impairment.

## 1 Introduction

Diabetes mellitus, a chronic metabolic disorder characterized by elevated blood glucose levels, poses a significant public health challenge globally. With approximately 537 million people living with diabetes worldwide, 24 million of these individuals reside in Africa. Visual Impairment (VI), a common and debilitating complication, is a significant concern for diabetic patients [1, 2]. The condition occurs when high blood sugar levels cause damage to the blood vessels in the retina, leading to vision problems. In Ethiopia, with diabetes prevalence ranging from 0.34% to 12.4%, the incidence of **VI** is of growing concern, as more individuals are at risk of developing this debilitating complication [3, 4]. **VI** is a serious eye condition caused by prolonged high blood sugar levels that damage the retinal blood vessels, leading to vision impairment or blindness. It is a significant complication of diabetes, affecting the ocular system and potentially causing substandard vision [5, 6]. Individuals with **VI** may also experience a loss of visual field, light sensitivity, double vision, and visual distortion, all of which can complicate the perception of images. As the condition progresses, these issues may significantly impact daily activities, including reading, vision, and recognizing faces. Early detection and management, including controlling blood glucose levels and monitoring eye health, are essential to prevent the worsening of these symptoms and preserve vision [7, 8].

Diabetic patients face substantial challenges due to visual impairment, with **VI** being a crippling problem that significantly impacts their quality of life. **VI** can lead to vision loss, limiting the ability to perform daily tasks such as reading, living, and recognizing faces. The progression of the disease can result in irreversible damage if not managed properly, making it a significant concern for diabetic individuals. Early detection, regular eye screenings, and proper management of blood glucose levels are crucial to preventing severe visual impairment from **VI** [9]. In turn, the impact of **VI** extends beyond vision loss, leading to higher unemployment rates, decreased productivity, and increased medical expenses. As daily living activities become more challenging, individuals with **VI** may experience mental health issues such as depression and anxiety, further affecting their overall well‐being. The loss of independence and inability to perform essential tasks can also contribute to social isolation, exacerbating the adverse effects on their quality of life. Consequently, **VI** poses a significant burden on both individuals and society, highlighting the need for effective prevention and management strategies [10, 11].

Globally, approximately 2.2 billion people suffer from near or distant visual impairment, with 3.9 million individuals affected by **VI**. This highlights the significant impact of **VI** on global eye health, particularly among individuals with diabetes. As a leading cause of preventable blindness, diabetic retinopathy contributes to the growing burden of visual impairment, emphasizing the need for early detection, effective management, and targeted interventions to reduce its prevalence and associated complications [12]. In Africa, the prevalence of visual impairment among diabetic patients can reach as high as 78.25%. This alarming statistic underscores the significant public health challenge posed by **VI** on the continent. The high prevalence highlights the urgent need for targeted prevention strategies, early diagnosis, and effective management of diabetes and its ocular complications to reduce the burden of visual impairment and improve the quality of life for affected individuals in Africa [13, 14, 15]. Different studies conducted in Ethiopia showed an incongruent prevalence of visual impairment among people with diabetes, ranging from 7.3 [16] to 70.06% [17]. A lack of glycemic control, poor physical exercise, older age, long duration of diabetes, and the type of treatment have all been found to increase the magnitude of **VI** [18, 19, 20].

VI is an escalating public health issue among diabetic patients, severely impacting their quality of life and placing increasing pressure on healthcare systems. In Ethiopia, multiple studies have documented widely varying prevalence rates and risk factors, leading to inconsistencies in understanding the determinants of VI. Although previous meta‐analyses have estimated the overall prevalence, there has been limited systematic investigation of its key predictors, which are presented in a separate random‐effects model, and further uncovers profound regional disparities, with prevalence [21]. The current findings offer valuable insights into regional disparities and risk factors associated with diabetes. Using a random‐effects model that combines various factors and pooled prevalence, the study highlights regional differences. This information is helpful in designing targeted interventions specific to each region. Such interventions could guide evidence‐based policy decisions and help alleviate the burden of diabetes‐related visual impairment in Ethiopia. We have also done precision estimation of both non‐modifiable (age) and modifiable (physical activity) risk factors. The precision of estimates varies between factors, with physical activity showing a narrower confidence interval, suggesting more consistent effects across studies. In contrast, age demonstrates wider confidence intervals, indicating greater variability in this association across different study populations. These findings highlight both non‐modifiable (age) and modifiable (physical activity) risk factors that should inform targeted clinical interventions and public health strategies for diabetic populations in Ethiopia.

## 2 Methods

### 2.1 Design and Searching Strategies

We conducted a comprehensive and systematic literature search to identify studies on the prevalence of risk factors for VI among diabetic patients in Ethiopia, published between 2013 and January 30, 2024. The search was performed across several databases, including PubMed, Google Scholar, Cochrane, CINAHL, Web of Science, and Scopus. Additionally, gray literature was sourced from online research repositories, and manual searches were conducted in key Ethiopian journals, including the Journal of Health Sciences, the Medical Journal, the Journal of Health and Development, and the Journal of Health and Biomedical Sciences. To ensure a thorough review, we also examined the reference lists of relevant articles to identify studies that were not captured in the initial database search. Using EndNote X7, the referencing manager, we downloaded, organized, and cited the articles. The search was conducted between January 1 and January 30, 2024, employing pre‐defined search terms developed based on the Medical Subject Headings (MeSH) and Boolean operators. These terms included “Visual impairment “OR” VI” AND “Risk Factors of Visual impairment “AND “Diabetic Patients” AND “Ethiopia.” The search strategy adhered to the Preferred Reporting Items for Systematic Reviews and Meta‐Analysis (PRISMA) guidelines to ensure transparency and reproducibility in the review process. This approach focused on studies that specifically assessed VI prevalence, regional disparities, and its risk factors among diabetic patients in Ethiopia [22].

### 2.2 Eligible Criteria

#### 2.2.1 Inclusion Criteria

The study specifically focused on identifying and analyzing the risk factors influencing VI among diabetic patients in Ethiopia. By examining cross‐sectional studies published between 2013 and January 30, 2024, the review aimed to pinpoint the various factors contributing to the prevalence of VI in this population. These risk factors included, but were not limited to, factors such as duration of diabetes, Insulin Treatment, poor glycemic control, hypertension, age, gender, family history of diabetes, and the presence of other comorbid conditions like hyperlipidemia. The review also considered the impact of socio‐economic factors, access to healthcare, and regional variations within Ethiopia, as these could potentially influence the incidence and progression of VI. By synthesizing data from multiple studies, the review sought to provide a comprehensive understanding of the key determinants of VI in Ethiopia, which could inform targeted public health interventions and management strategies for diabetic patients at risk of developing retinopathy.

#### 2.2.2 Exclusion Criteria

In this study, we excluded all citations that did not include an abstract or full text, as these were deemed insufficient for the systematic review. Additionally, articles written in languages other than English were excluded to maintain consistency and accessibility. This decision was made to ensure that all included studies could be comprehensively reviewed and understood by the research team, as none of the reviewers were fluent in languages other than English. Furthermore, non‐English articles would have required additional resources for translation, which was not feasible within the scope and timeline of the review. Studies that did not focus on the risk factors influencing VI in Ethiopia were also excluded, ensuring that the review specifically addressed the determinants of VI within the Ethiopian context. This approach helped narrow the scope of the research to only those studies that directly contributed to understanding the risk factors of VI among diabetic patients in Ethiopia, thereby enhancing the relevance and accuracy of the findings.

### 2.3 Outcome Measures

This systematic review and meta‐analysis have three primary objectives. The first objective is to determine the pooled prevalence of VI among diabetic patients in Ethiopia. VI is defined as a microvascular complication of diabetes, affecting the retina, with varying degrees of severity, ranging from mild non‐proliferative VI to more advanced stages such as proliferative VI and diabetic macular edema. The second objective is to identify and analyze the risk factors influencing the development and progression of VI in Ethiopian diabetic patients. These risk factors include factors such as the duration of diabetes, glycemic control, hypertension, dyslipidemia, age, gender, family history of diabetes, and the presence of other comorbid conditions, as well as regional disparities. By focusing on these risk factors and regional inequalities, this review seeks to provide a comprehensive understanding of the determinants of VI in Ethiopia, which can inform public health strategies and clinical interventions aimed at reducing the burden of VI among the diabetic population [23].

### 2.4 Data Extraction and Quality Appraisal

The studies retrieved from all databases were imported into EndNote X7, where duplicates were identified and removed. Two groups of review authors reviewed all papers: group one (C.Y. & A.G.Y.), who first reviewed the abstracts and titles, and then the full texts—in the event of disagreements between the review authors, CY and AGY initially attempted to resolve these issues through discussion and consensus. If a consensus could not be reached, the reviewer (Y.A.) was then involved to resolve the dispute. Two authors (C.Y. and A.G.Y.) independently extracted all the pertinent data using a standardized data extraction template. The parameters in the data extraction template included the author's name, sample size, study area, study design, year of publication, OR, and 95% CI. The JBI critical appraisal checklist encompasses a comprehensive set of criteria, including the appropriateness of the study design, the sampling method, the measurement of exposure and outcome variables, and the statistical analysis. Each study was assessed for methodological quality across these key areas, and a score was assigned based on how well it met the established criteria [24]. Finally, only articles that achieved a quality assessment score of 50% or more on the JBI critical appraisal checklist were included in the review. This threshold was set to ensure that the studies included in the analysis met a minimum standard of methodological rigor, reducing the risk of bias and enhancing the reliability of the findings. Studies that scored below 50% were excluded, as they were considered to have significant methodological weaknesses that could undermine the validity of the results [22].

### 2.5 Data Analysis

RevMan software 5.4 and R statistical software (version 4.5.1) were used to analyze data extracted from Microsoft Excel. Heterogeneity across studies was assessed using the *I*² test, with *I*² values interpreted as moderate (30%–60%), substantial (60%–90%), and considerable (90%–100%) heterogeneity. Given the expected clinical and methodological diversity among included studies, all meta‐analyses were conducted using random‐effects models, which account for between‐study variations and provide more conservative and generalizable estimates. The DerSimonian and Laird method was used to calculate pooled effects for all analyses, offering more accurate and reliable pooled forecasts in the presence of heterogeneity [25].

To identify the sources of heterogeneity and explore regional disparities, we conducted comprehensive subgroup analyses based on geographic region (Amhara, SNNP, Addis Ababa, Oromia, Tigray), publication year, and sample size. For the regional disparities analysis, forest plots were generated to visually represent prevalence variations across different geographic regions of Ethiopia, providing insights into spatial patterns of visual impairment burden. Additionally, temporal trends were analyzed by comparing pre‐2020 and post‐2020 studies to identify evolving patterns in VI prevalence. Meta‐analyses generated pooled prevalence estimates and odds ratios, with results visually presented through forest plots to assess variation across studies and guide interpretation of the risk factors influencing VI in Ethiopia. Publication bias was evaluated both subjectively using funnel plots and objectively through Egger's weighted correlation and Begg's regression intercept tests. These methods helped evaluate the symmetry of the funnel plots and provided statistical evidence regarding the presence of publication bias in the included studies [26, 27]. To ensure robust findings, sensitivity analyses were performed by sequentially excluding each study to assess the stability of the pooled estimates.

The regional disparities analysis specifically aimed to identify geographic variations in the prevalence of visual impairment, which could inform targeted public health interventions and resource allocation across different regions of Ethiopia. All statistical tests were two‐sided, with *p* < 0.05 considered statistically significant.

## 3 Results

### 3.1 Characteristics of the Study

**Figure 1:**
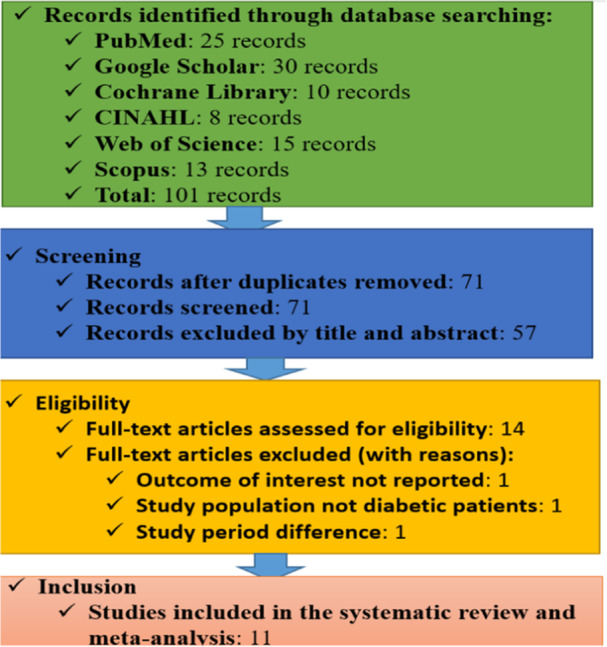
Flowchart diagram illustrating the selection of studies for the systematic review and meta‐analysis of prevalence, regional disparities, and risk factors of visual impairment among diabetic patients in Ethiopia, 2024.

The systematic review and meta‐analysis followed the PRISMA guidelines to ensure a transparent and replicable selection process. A total of 101 records were identified across six databases (PubMed: 25, Google Scholar: 30, Cochrane Library: 10, CINAHL: 8, Web of Science: 15, Scopus: 13), with an additional three records sourced externally. After removing duplicates, 71 unique records were screened, and 57 were excluded based on title and abstract relevance. Fourteen full‐text articles were assessed for eligibility, with three excluded due to irrelevance to the outcome, non‐diabetic study populations, or differences in the study period. Ultimately, 11 studies met the inclusion criteria and were analyzed (Figure 1).

### 3.2 Description of the Included Studies

Table 1 presents a summary of the 11 primary studies included in this systematic review and meta‐analysis, which involved a total of 4,204 diabetic patients across various regions of Ethiopia. These studies, published between 2013 and 2022, all utilized a cross‐sectional design and varied in sample size, ranging from 225 to 881 participants. The studies were conducted in multiple regions, with the majority (6 studies) from the Amhara region, followed by two from the Southern Nations, Nationalities, and Peoples’ (SNNP) region, and one each from Oromia, Addis Ababa (AA), and Tigray. The prevalence of VI among diabetic patients varied widely, from 7.3% to 70.06%, reflecting regional and population differences. All included studies had a quality score of 50% or higher, with most scoring between 75% and 87.5%, suggesting moderate to high‐quality evidence. This detailed breakdown offers key insights into the diversity of the studies, the varying prevalence of VI, and the regional differences across Ethiopia, which is crucial for understanding the broader context of visual impairment among diabetic patients in the country (Table 1).

**Table 1:** Characteristics of studies included in the systematic review and meta‐analysis of prevalence, regional disparities, and risk factors of visual impairment among diabetic patients in Ethiopia, 2024.

Author	Year	Region	Study design	Population	Sample size	Response rate (%)	Prevalence rate (%)	Quality score (%)
Alemayehu et al. [28]	2022	Southern	Cross‐sectional	DM‐patients	398	98.2	28.6	75
Seid et al. [29]	2020	Amhara	Cross‐sectional	DM‐patients	332	97	37.58	87.5
Mersha et al. [30]	2021	Amhara	Cross‐sectional	DM‐patients	296	95.2	39.5	87.5
Demilew et al. [31]	2020	Amhara	Cross‐sectional	DM‐patients	388	92	29.38	75
Cherinet et al. [17]	2018	AA	Cross‐sectional	DM‐patients	881	97.4	30	75
Asemu et al. [32]	2021	Tigray	Cross‐sectional	DM‐patients	401	96.3	70.06	87.5
Tilahun et al. (34)	2020	Amhara	Cross‐sectional	DM‐patients	302	93.5	37.7	87.5
Siersma et al. (35)	2019	SNNP	Cross‐sectional	DM‐patients	241	91	15.4	87.5
Assefa et al. (36)	2020	Amhara	Cross‐sectional	DM‐patients	416	98.6	16.82	75
Ejigu et al. (37)	2021	Amhara	Cross‐sectional	DM‐patients	225	94.5	10.7	75
Sharew et al. [16]	2013	Oromia	Cross‐sectional	DM‐patients	324	91.7	7.3	75

### 3.3 Meta‐Analysis on the Pooled Prevalence of VI Among Diabetic Patients in Ethiopia

**Figure 2:**
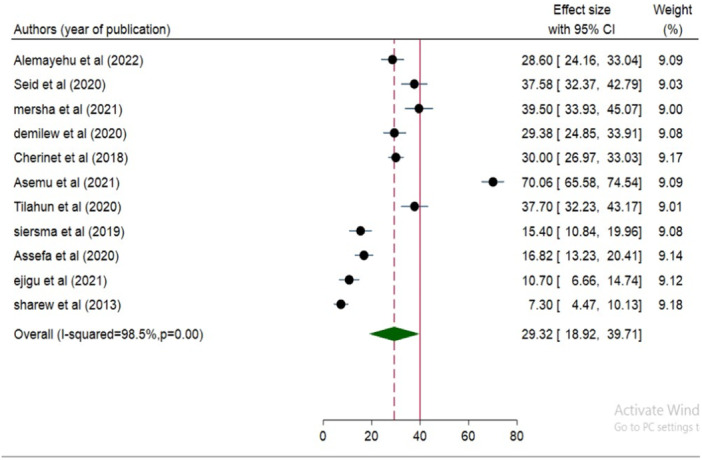
Forest plot of the pooled prevalence of VI among diabetic patients in Ethiopia, 2024.

Based on 11 studies involving 4204 diabetic patients, the random effect pooled prevalence of **VI** among diabetic patients in Ethiopia was 29.32% (95% CI: 18.92, 39.71). As shown by the I2 (variation in ES attributable to heterogeneity) test result, there was substantial heterogeneity across the studies, with *I* ^2^ = 98.5%, at *p* = 0.001. Therefore, the pooled prevalence of **VI** was estimated using random‐effects models. Results were displayed as forest plots (Figure 2).

## 4 Publication Bias

**Figure 3:**
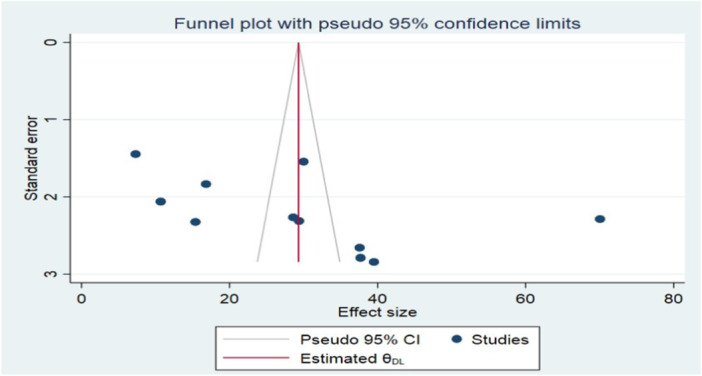
Funnel plot of the studies included in a systematic review and meta‐analysis of prevalence, regional disparities, and risk factors of visual impairment among diabetic patients in Ethiopia, 2024.

**Figure 4:**
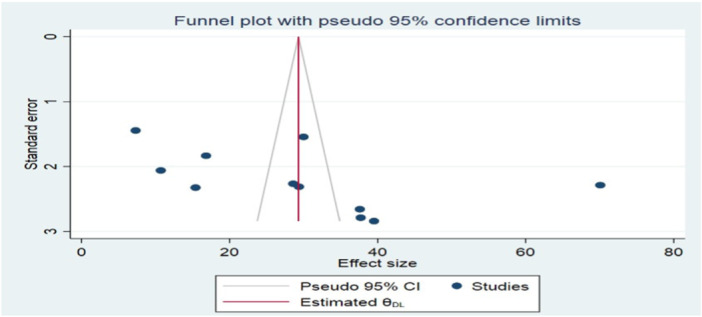
Egger's test of the studies included in a systematic review and meta‐analysis of prevalence, regional disparities, and risk factors of visual impairment among diabetic patients in Ethiopia, 2024.

By examining the funnel plot and performing Begg's and Egger's tests, publication bias was assessed visually and statistically. An inverted funnel with a symmetrical distribution of the publications indicated no publication bias (Figure 3). Additionally, Begg and Egger's tests confirmed no publication bias existed among the studies included to estimate the pooled prevalence of visual impairment with p‐values of (*p* = 0.563 and 0.098) (Figure 4).

## 5 Subgroup Analysis

**Figure 5:**
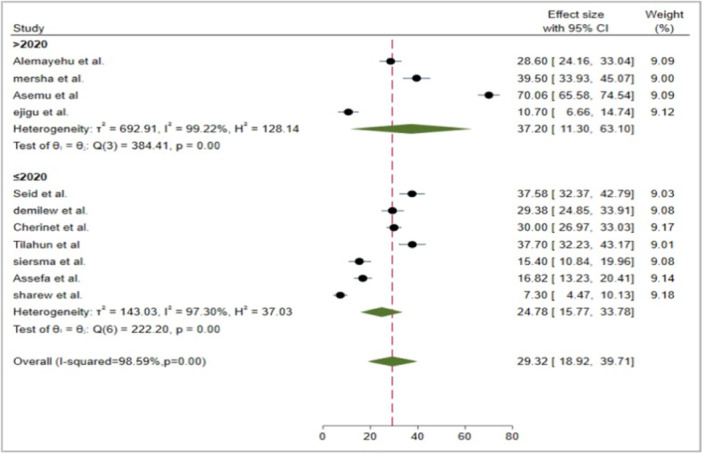
Forest plot of the pooled prevalence of VI among diabetic patients in Ethiopia by publication year, 2024.

Studies conducted after 2020 show substantial variability in effect sizes, with prevalence estimates ranging from very low to relatively high (10.70% to 70.06%). For example, studies like Alemayehu et al. (28.60%) and Asemu et al. (39.50%) report moderate effect sizes, while others, such as Ejigu et al., present a notably high estimate of 70.06%. The heterogeneity in this group is exceptionally high (*I*² = 99.22%), suggesting significant variability in findings. This variability may be attributed to differences in study populations, methodologies, or geographical locations, which could explain the differing outcomes across these studies. Despite this variability, the pooled prevalence estimate for studies published after 2020 is 37.20% (95% CI: 11.30%, 63.10%). In contrast, studies published before 2020 exhibit relatively more consistent results, with effect sizes ranging from 7.30% (Sharew et al.) to 37.58% (Seid et al.). The study by Sharew et al. reports the lowest prevalence of VI, while studies like Seid et al. and Tilahun et al. present higher prevalence estimates, ranging from 30% to 37%. The heterogeneity in this group (*I*² = 97.30%) remains high, indicating considerable variability in findings, although it is less pronounced compared to the post‐2020 group. The pooled prevalence for studies conducted before 2020 is 24.78% (95% CI: 15.77%, 33.78%) (Figure 5).

**Figure 6:**
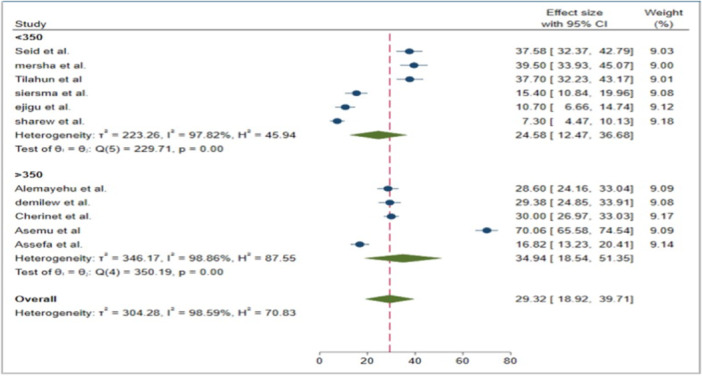
Forest plot of the pooled prevalence of prevalence, regional disparities, and risk factors of visual impairment among diabetic patients in Ethiopia, 2024.

Similarly, a subgroup analysis based on sample size was conducted to identify any differences in prevalence. Accordingly, the pooled prevalence was 34.94% (95% CI: 18.54, 51.35) for studies with large sample sizes ( > 350), but decreased for studies with lower sample sizes ( < 350), with a pooled prevalence of 24.58% (95% CI: 12.47, 36.68) (Figure 6).

### 5.1 Geographic Variation in Visual Impairment Prevalence Among Diabetic Patients in Ethiopia

**Figure 7:**
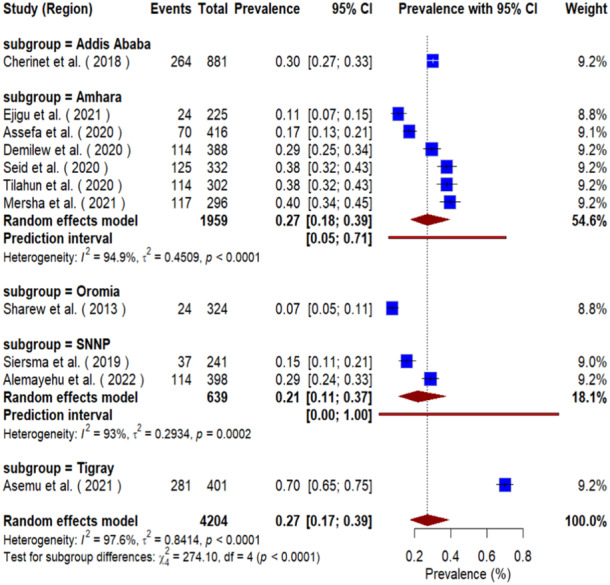
Forest plot of prevalence estimates with subgroup analysis by geographic region of VI among diabetic patients in Ethiopia.

The forest plot reveals a statistically significant difference in the prevalence of visual impairment among diabetic patients across different geographic regions of Ethiopia (Test for subgroup differences: χ² = 274.10, df = 4, *p* < 0.0001). The overall pooled prevalence was 27% (95% CI: 0.17 to 0.39), but this masked considerable regional heterogeneity (*I*² = 97.6%). The prevalence was significantly highest in the Tigray region at 70% (95% CI: 0.65–0.75), which does not overlap with the confidence intervals of other areas, indicating a significantly higher burden. In contrast, the Oromia region had a significantly lower prevalence of 7% (95% CI: 0.05–0.11). The Amhara and SNNP regions showed intermediate, yet variable, pooled estimates of 27% (95% CI: 0.18–0.39) and 21% (95% CI: 0.11–0.37), respectively, though with high within‐subgroup heterogeneity. The non‐overlapping confidence intervals, particularly between the extremes of Tigray and Oromia, provide strong evidence for a genuine and significant geographic disparity in the prevalence of visual impairment (Figure 7).

### 5.2 Risk Factors of VI Among Diabetic Patients in Ethiopia

**Figure 8:**
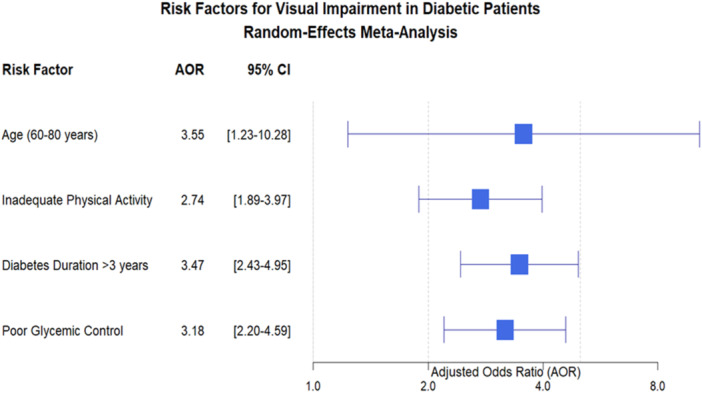
Forest plot displaying Adjusted Odds Ratios (AOR) with 95% Confidence Intervals (CI) for risk factors associated with visual impairment among diabetic patients in Ethiopia.

The plot illustrates four significant risk factors identified through a random‐effects meta‐analysis of 11 studies involving 4,204 participants. Each risk factor is represented by a diamond‐shaped point estimate with horizontal error bars indicating the 95% CI. The vertical reference line at AOR = 1.0 represents no association. All risk factors demonstrate statistically significant associations with visual impairment, as evidenced by 95% CIs that do not cross the null value of 1.0. The meta‐analysis revealed four considerable risk factors for visual impairment among diabetic patients in Ethiopia, all with statistically significant associations (95% CIs excluding 1.0). Older age (60–80 years) showed the strongest association with visual impairment (AOR = 3.55, 95% CI: 1.23–10.28), indicating that elderly diabetic patients had over 3.5 times higher odds of developing visual complications compared to younger patients. Diabetes duration exceeding 3 years was also strongly associated (AOR = 3.47, 95% CI: 2.43–4.95), demonstrating that prolonged disease duration substantially increases visual impairment risk. Poor glycemic control (AOR = 3.18, 95% CI: 2.20–4.59) and inadequate physical activity (AOR = 2.74, 95% CI: 1.89–3.97) were additional significant modifiable risk factors, with approximately three‐fold and 2.7‐fold increased odds of visual impairment, respectively. The precision of these estimates varies across factors, with diabetes duration and glycemic control showing narrower confidence intervals, suggesting more consistent effects across studies. In contrast, age demonstrates wider confidence intervals, indicating greater variability in this association across different study populations (Figure 8).

## 6 Discussion

The VI is one of the most debilitating challenges worldwide. Thus, this systematic review and meta‐analysis aimed to estimate the pooled prevalence of VI and risk factors among diabetic patients in Ethiopia.

According to this meta‐analysis, the overall pooled prevalence of VI among people with diabetes in Ethiopia was 29.32% (95% CI: 18.92, 39.71). This finding was consistent with a study done in Cameroon (29.7%) [14]. The consistency of findings across regions may be attributed to similar underlying health factors, such as comparable healthcare access and diagnostic methods, which help standardize results despite regional differences. However, this pooled prevalence was found to be lower than a study conducted in Yemen (55%) [33]. The discrepancy might be due to the difference in the study population, as in Yemen, diabetes patients undergoing ophthalmic evaluation at an eye care clinic and already experiencing compliant visual symptoms were the study subjects. The magnitude of VI would be exacerbated if the prevalence were studied in this setting. Also, the current finding was higher than the studies conducted in Malawi (5%) [13], Tunisia (17.8%) [28], Ghana (18.4%) [20], Turkey (13.5%) [19], and China (3.6%) [18]. This might be due to the way they operationalize the outcome variable. Unlike our study and others, their study exploits best‐corrected visual acuity while classifying visual impairment.

The pooled prevalence of VI among people with diabetes in studies conducted before 2020 was 24.78%, while it was 37.20% in studies conducted after 2020. A shift in lifestyle from a fumigated to a cognizant one may explain the relative increase between previous and recent studies.

In line with other studies conducted in Tunisia [28] and Turkey [19] In this study, age is significantly associated with VI among people with diabetes. That is, diabetic patients aged 60–80 years were at higher odds of VI as compared to their counterparts. This might be because older individuals with diabetes often have a longer duration of the disease [29]. The longer someone has diabetes, the higher the likelihood of developing complications. Besides, Older individuals with diabetes may also have other health conditions, such as hypertension and cardiovascular disease. These conditions, along with diabetes, can contribute to a higher risk of eye problems.

The findings from this systematic review and meta‐analysis revealed that physical exercise is another prominent variable positively associated with VI. Diabetic patients with poor physical exercise habits were more likely to have visual impairment than their counterparts. This finding is supported by a study conducted in China [30]. Such a tie‐in might be because persisting good physical exercise habits is pivotal for diabetic patients to handle their condition effectively and lessen the risk of burdens, including VI [31]. Regular exercise provides better blood sugar control, cardiovascular health, weight management, and overall well‐being, all of which play a pivotal role in preventing diabetic complications that affect the eyes.

Moreover, in this study, participants with a 7–9‐year duration of diabetes were more likely to have visual impairment than their counterparts. This is supported by a study done in Tunisia [28]. and Ghana [20] This might be due to the cumulative effects of prolonged exposure to high blood sugar levels, chronic inflammation, oxidative stress, and associated complications, which contribute to an increased risk of VI in individuals with a longer duration of diabetes.

Furthermore, in this meta‐analysis, glycemic control exercise is another prominent variable positively associated with VI. That is, diabetic patients who had poor glycemic control were more likely to be VI than their counterparts. This finding is supported by a study conducted in China [18]. Such a tie‐in could be because high blood sugar levels over an extended period can lead to impairment of small retinal blood vessels [32].

Based on the findings of this systematic review and meta‐analysis, we propose a conceptual framework that emphasizes the complex interaction between demographic, behavioral, and clinical factors in influencing VI among diabetic patients. The framework highlights key risk factors, such as age, with older patients (60–80 years) being at higher risk due to the longer duration of diabetes and associated comorbidities like hypertension and cardiovascular disease. It also incorporates lifestyle factors, such as inferior physical exercise habits, which exacerbate the risk of VI by hindering the effective management of blood sugar and overall health. Furthermore, the framework underscores the role of glycemic control, with poor control significantly increasing the risk of retinal damage and, consequently, VI. By integrating these interconnected variables, the conceptual model offers a comprehensive approach to understanding and addressing the risk of visual impairment in diabetic populations, suggesting that targeted interventions considering age, lifestyle modifications, and glycemic control could significantly reduce the burden of VI. This framework not only aids in improving clinical strategies but also paves the way for future research to explore more precise interventions for preventing and managing VI in diabetic patients.

### 6.1 Strengths and Limitations of the Study

This study has both strengths and limitations. A key strength is that it is the first systematic review and meta‐analysis to estimate the regional pooled prevalence disparities of VI and combine risk factors in the random effects forest plot among diabetic patients in Ethiopia. However, it is essential to note that while these models adjust for variability, they do not entirely eliminate the impact of all potential confounders, such as socioeconomic or healthcare access variables, which may influence the observed associations. A more explicit assessment of these confounding factors would have further strengthened the interpretation of the results and improved the critique of the findings. However, the study has limitations, including its selective inclusion of high‐quality studies and lack of coverage of all Ethiopian regions, which may affect the representativeness of the findings. The limitation to English‐scripted studies also reduced the number of included studies, potentially impacting the comprehensiveness of the analysis. While the funnel plot provides a visual representation of publication bias, visual inspection alone may be subjective. To improve accuracy, the Trim‐and‐Fill method is better suited for a more rigorous evaluation of publication bias. Furthermore, the inclusion of only cross‐sectional studies limits the ability to infer causal relationships between risk factors and visual impairment. While cross‐sectional studies provide valuable insight into the associations between risk factors and VI, they do not establish temporal or cause‐and‐effect relationships. Longitudinal or cohort studies, if available, could have strengthened the findings by providing a clearer understanding of how risk factors influence the development of visual impairment over time.

## 7 Conclusion and Recommendation

Conclusion This systematic review and meta‐analysis confirm that VI represents a significant and widespread complication of diabetes mellitus within Ethiopia, with an overall pooled prevalence of nearly one in three patients. The analysis further reveals profound geographic disparities, indicating that the burden of disease is not uniformly distributed across the country's regions, but is instead influenced by distinct regional factors. The identification of key modifiable risk factors, specifically advanced age, prolonged diabetes duration, physical inactivity, and poor glycemic control, provides a clear and actionable framework for intervention. The considerable heterogeneity observed underscores the complex interplay of clinical, behavioral, and potentially unmeasured socioeconomic or healthcare access variables in the development of VI. This evidence collectively highlights VI as a critical public health challenge that jeopardizes the quality of life of diabetic individuals and imposes a significant burden on the Ethiopian healthcare system.

Recommendation To effectively mitigate the burden of visual impairment among diabetic patients in Ethiopia, a multifaceted and collaborative strategy is urgently required. The following actions are recommended:

1. Integrated and Targeted Clinical Care:
  Strengthen Diabetes Management: Prioritize tight glycemic control through patient education, regular monitoring of HbA1c, and ensuring consistent access to essential medications.
  Implement Routine Eye Screening: Establish and enforce national guidelines mandating annual or biennial dilated eye examinations for all diabetic patients, with a focus on early detection of retinopathy.
  Develop Regional Protocols: Create and resource region‐specific intervention plans to address the stark disparities, ensuring that high‐burden regions, such as Tigray, receive intensified screening and treatment resources.
2. Public Health and Community‐Based Interventions:
  Promote Lifestyle Modifications: Launch public health campaigns emphasizing the crucial role of regular physical activity in preventing diabetic complications, including VI.
  Enhance Patient Education: Develop and disseminate culturally appropriate educational materials that empower patients to understand the risk factors for VI and the importance of adherence to treatment and screening schedules.
3. Health System Strengthening:
  Build Capacity: Train healthcare workers, including nurses and health officers, in the fundamentals of diabetic eye care to facilitate task‐shifting and expand service delivery, especially in primary healthcare settings.
  Improve Access to Specialized Care: Invest in diagnostic equipment and build referral pathways to ensure that patients diagnosed with sight‐threatening VI can access timely ophthalmological care.
4. Future Research and Policy:

Conduct Longitudinal Studies: Fund research to establish causal relationships and understand the long‐term progression of VI in the Ethiopian context.

Investigate Disparities: Further research is needed to elucidate the underlying drivers of the dramatic regional variations, exploring factors such as healthcare infrastructure, environmental influences, and genetic predispositions.

Integrate NCD and Eye Health: Policymakers should integrate diabetic eye care into the broader Non‐Communicable Disease (NCD) strategy and ensure sustainable financing for these essential services.

By adopting a comprehensive approach that bridges clinical practice, public health initiatives, and health system reform, Ethiopia can make significant strides in reducing the incidence of blindness and visual impairment caused by diabetes, thereby preserving vision and improving the overall well‐being of its population.

## Author Contributions

**Chalachew Yenew:** conceptualization, investigation, methodology, writing – original draft, writing – review and editing, visualization, validation, formal analysis, supervision, data curation. **Yehzibalem Azmeraw:** conceptualization, visualization, software, writing – original draft, writing – review and editing. **Almaw Genet Yeshiwas:** conceptualization, investigation, methodology, visualization, writing – original draft, writing – review and editing. all authors have read and approved the final version of the manuscript.

## Funding

The authors received no specific funding for this work.

## Conflicts of Interest

The authors declare no conflicts of interest.

## Transparency Statement

This systematic review and meta‐analysis were conducted in accordance with the PRISMA (Preferred Reporting Items for Systematic Reviews and Meta‐Analyses) guidelines. A comprehensive literature search was performed using predefined eligibility criteria, and multiple reviewers independently conducted data extraction to ensure accuracy. Statistical analyses, including a random‐effects meta‐analysis, were conducted using appropriate methodologies. All included studies are cited, and the data supporting the findings of this study are available upon reasonable request. There are no conflicts of interest to declare.

## Data Availability

The data that support the findings of this study are available in the supplementary material of this article.
